# Current Trends in Bone Tissue Engineering

**DOI:** 10.1155/2014/865270

**Published:** 2014-04-06

**Authors:** Marco Mravic, Bruno Péault, Aaron W. James

**Affiliations:** Department of Pathology & Laboratory Medicine, and Orthopedic Hospital Research Center, University of California, David Geffen School of Medicine, UCLA, 10833 Le Conte Avenue, A3-251 CHS, Los Angeles, CA 90095, USA

## Abstract

The development of tissue engineering and regeneration constitutes a new platform for translational medical research. Effective therapies for bone engineering typically employ the coordinated manipulation of cells, biologically active signaling molecules, and biomimetic, biodegradable scaffolds. Bone tissue engineering has become increasingly dependent on the merging of innovations from each of these fields, as they continue to evolve independently. This foreword will highlight some of the most recent advances in bone tissue engineering and regeneration, emphasizing the interconnected fields of stem cell biology, cell signaling biology, and biomaterial research. These include, for example, novel methods for mesenchymal stem cell purification, new methods of Wnt signaling pathway manipulation, and cutting edge computer assisted nanoscale design of bone scaffold materials. In the following special issue, we sought to incorporate these diverse areas of emphasis in order to reflect current trends in the field.

## 1. Introduction

The development of tissue engineering and regeneration constitutes a new platform for translational medical research. Effective therapies for bone engineering typically employ the coordinated manipulation of cells, biologically active signaling molecules, and biomimetic, biodegradable scaffolds. Bone tissue engineering has become increasingly dependent on merging innovations from each field, as they continue to evolve independently. Given the complexity and diverse nature of these research areas—from osteoprogenitor cell biology to biomaterials—a summary that fully encompasses the advances in bone tissue engineering is not possible. Instead, this foreword will examine some of the most recent advances in bone tissue engineering and regeneration, emphasizing the interconnected fields of cell biology, signaling biology, and biomaterial research.

## 2. Purified, Autologous Stem Cells

Tissue engineering efforts using autologous adult mesenchymal stem cell (MSC) sources such as cryopreserved umbilical cord blood, bone marrow, and adipose tissue have shown considerable ability to regenerate bone tissue. However, currently used sources of MSC populations require cultural expansion or selection by plastic adherence before they are effective or available for regenerative therapies. Phenotypic changes resulting from exposure to* in vitro *conditions are not well understood, and such changes can lead to negative therapeutic consequences such as reduced proliferation rate and significant morphological changes [1]. Notably, the former has been correlated with reduced bone formation in cultured human bone marrow MSC (bmMSC), using an ectopic mouse model [2]. Similarly, cord blood MSC require expansion, while plastic adherence is used to isolate multipotent MSC from adipose, comprising a portion of adipose-derived stromal cells (ASC). Despite the relatively high stem cell frequency in adipose, the heterogeneity of the uncultured stromal vascular fraction (SVF) restricts its effectiveness as a freshly isolated MSC source. Overall, even MSC freshly isolated or at early passages are a heterogeneous group of cells [3, 4].

Thus, cutting edge efforts have attempted to purify MSC population, most often using fluorescence activated cell sorting (FACS) by differential expression of cell surface markers. Choosing markers that distinguish MSC for multipotency and regenerative properties is an area of avid and ongoing research. One step forward is the identification of a superior subset of adipose-derived stromal cells for bone regeneration. Levi et al. reported a subset of ASC with low expression of the MSC marker CD105 (Endoglin) [5] as an enriched osteoprogenitor cell population with the downregulation of TGF-*β*1 signaling. In a similar attempt to purify ASC populations, Chung et al. selected for CD90 (Thy-1) expression to obtain a purified ASC subset population with enhanced osteogenic potential [6]. These studies highlight how even a single cell surface marker may be used in order to purify a more potent progenitor cell population for bone tissue engineering. Similar approaches have used diverse surface markers to enhance for a given attribute or phenotype of MSC. These include, for example, selecting for CD34^+^ ASC for higher cell proliferation [7, 8], CD34^+^ CD90^+^ ASC for improved vasculogenesis [9], and CD105^+^ ASC for heightened chondrogenic potential [10, 11].

In another, not wholly dissimilar, tactic-MSC purification has been performed via the deduction of the perivascular origin of MSC [12–18]. Having found that MSC reside in a perivascular distribution, investigators have attempted to purify MSC by FACS mediated isolation of cells with perivascular markers from several organs. These cells, termed perivascular stem cells (PSC), encompass both pericytes (CD146^+^, CD34^−^, CD45^−^, and CD56^−^) and adventitial cells (CD34^+^, CD31^−^, and CD146^−^) [12, 19]. As PSC are abundant in adipose, a highly vascular tissue, their purification obviates the need and complication of cultural expansion or* in vitro* selection. Additionally, PSC have exhibited expression of MSC markers and multilineage multipotency* in vitro* while enhancing both bone formation and bone repair* in vivo*, as compared to unsorted SVF in mouse models [19, 20]. Thus, adipose-derived PSC are an abundantly available autologous source of safe and effective MSC progenitors. A further review of perivascular MSC isolation by flow cytometry is available [14] and reviews of PSC and pericytes' ability to regenerate or naturally generate other tissues are also available [21–23].

Unfortunately, the use of culture expansion and fluorescently label antibodies for flow cytometry manipulates the cells and introduces animal products, warranting the need for clinical trials and complicating regulatory approval. Thus, such methods for cell therapies require FDA regulation as well as the expense of following Good Manufacturing Practices for antibodies and biologics used. Although still in its infancy, label-free sorting using microfluidic technologies may provide an alternative route to purifying multipotent cells from mature tissue for autologous cell therapies. For example, adrenal cortical progenitor cells were enriched for using label-free size-based inertial ordering, removing their differentiated counterparts in a microfluidic chip [24]. As nuclear deformability has been studied extensively as a marker of pluripotency [25], similar sterile fluid flow devices have incorporated hydrodynamic stretching to confirm this characteristic in human and murine embryonic stem cells and their differentiated counterparts [26, 27]. Thus, mechanophenotyping cells from tissues containing MSC may elucidate biophysical criteria for isolating mesenchymal cells enriched for multipotency and regenerative properties. Subsequent purification of such tissue digests based on size and deformability, whether by active or passive sorting techniques, may remove debris and unwanted, terminally differentiated cells while yielding these enriched subsets. With such biophysical sorting methods in development, it is possible that FDA licensure for minimal manipulation may be more easily obtainable for bone therapies.

Despite the emergence of biomarker-free sorting methods, molecular criteria are the current gold standard for stem cell purification. Since safe isolation of abundant and osteocapable cell sources has been accomplished, current efforts focus on identifying surface and intracellular markers indicative of high osteogenic potential as well responsiveness to osteoinductive signals. Nevertheless the purification of MSC for those with enhanced osteogenic potential has been attempted from diverse avenues by multiple investigative groups-showing robust, promising results.

## 3. Manipulation of Signaling Pathways and Differentiation

A commonly studied method of promoting osteogenic differentiation is to manipulate signaling pathways important in skeletal development, such as Wnt [28], Hedgehog [29–31], BMP (Bone Morphogenic Protein) [32–34], and the emerging anti-inflammatory molecule NELL-1 [30, 35–37]. A transition away from an interest in BMP2 may be expected due to an increasing side effect profile including postoperative inflammation [35, 38] and osteoclast activation [39–41]. The FDA has reported on safety concerns concerning BMP2 use [42, 43].

Of the above-mentioned signaling pathways, perhaps none is more intensely studied than components of the Wnt pathway for the treatment of osteoporotic bone loss. Romosozumab, a neutralizing antibody against Sclerostin, an inhibitor of Wnt coreceptor LRP (low density lipoprotein receptor-related protein) has shown to mimic high bone mass disease sclerosteosis, enhancing osteoblastic differentiation and bone formation [44, 45]. Amgen Inc. is currently conducting Phase III clinical trials using this antibody to treat postmenopausal osteoporosis [46]. Romosozumab joins Amgen's anti-RANK antibody Denosumab as FDA approved osteoporosis treatments, all of which could be pursued for tissue engineering applications. Similarly, a neutralizing antibody against Wnt inhibitor Dkk1 (Dickkopf1), which is in Phase I/II clinical trials for multiple myeloma, has been observed to have anabolic bone activity [47]. Recently, tyrosine kinase inhibitor dasatinib, the FDA approved cancer drug Sprycel, was shown to stimulate Wnt signaling and inhibit PDGFR-*β* (platelet derived growth factor receptor-*β*) and c-Src phosphorylation, promoting osteoblast differentiation and inducing an anabolic bone effect* in vitro* and* in vivo* while inhibiting osteoclast formation in hematopoietic progenitor cells* in vitro* [48]. Another interesting approach, coating the surface of hydrophilic titanium scaffolds with Wnt agonist lithium chloride, via GSK3 inhibition, was shown to increase bone density, independent of the scaffold [49]. This approach exemplifies coordinated delivery of developmental signaling modulation and biomimetic materials.

Manipulating expression and differentiation at the genetic level also allows for potentially more closely orchestrated control of cellular and tissue phenotype. Micro-RNAs (miRNAs), small noncoding RNA involved in transcriptional regulation, have recently been targeted to enrich bone regeneration. Enhanced bone formation and vascularization were observed upon delivery of miRNA 26a in both subcutaneous and cranial repair mouse models [50]. Likewise, transfection of MSC with mimics and inhibitors of miRNAs 148 and 489 increased* in vitro* osteogenesis, evaluated by calcium deposition and gene expression [51]. Comprehensive reviews are available for miRNA in bone development and regeneration [52–54]. Likewise, with the development of safer, nonviral transfection agents, gene therapy via BMPs [55] and other growth factors have been used to supplement bone reconstruction. Moreover, nonviral vectors embedded in biodegradable scaffolds, termed gene-activated matrices, allows for gradual and sustained delivery of a gene postoperatively [56]. Bone tissue engineering and regenerative therapies rely on speeding tissue differentiation and controlling morphology by targeting miRNA, introducing genes and recombinant proteins and modulating developmental signaling pathways.

## 4. Use of Biomaterials

Design of biocompatible scaffolds for bone tissue engineering requires the balance of an osteoinductive cellular microenvironment, diffusion of soluble factors, flexibility, and mechanical loading appropriate for the anatomical site [57, 58]. Although there are limits on vascularization and innervation in whole organ reconstruction, recent advances in 3D printing (3D-P) provide a diverse source of scaffolds for bone tissue engineering. Tamjid et al. controlled properties, such as adherence, proliferation, and uniform tissue growth rate, of MCT3T-E1 preosteoblasts within the pores of indirectly 3D-printed polycaprolactone scaffolds by mimicking extracellular matrix (ECM) architecture with hydrophilic additives, including titania ceramic nanoparticles and bioglass microparticles particles [59]. In a similar attempt, porous alginate hydrogels amalgamated with gelatin microspheres loaded with BMP-2 were constructed with 3D-P and were used to gradually release BMP-2 to goat MSC* in vitro* [60]. Another freeform fabrication technique, laser microstereolithography (L-MSTL), fabricates 3D structures by selectively curing photopolymer on a moving platform layer by layer [61, 62]. In a recent study, L-MSTL was used to embed BMP-2 within poly(DL-lactic-co-glycolic acid) (PLGA) microspheres on a poly(propylene fumarate) photopolymer, which enhanced MT3T-E1 cell differentiation* in vitro* and outperformed both unloaded scaffolds and scaffolds made by the particulate leaching/gas foaming method in an rat cranial injury repair model [63]. Overall, computer assistant nanoscale design of biomimetic ECM, while still being in relative infancy of preclinical investigation, has potential to create a biomaterials fabrication platform for improved bone tissue engineering and regeneration.

Such meticulous design of tissue engineered constructs is necessary to allow for selective diffusion of biological molecules as well as migration and patterning of regenerative cells. Unfortunately, accurate* in vivo *prediction of the biological consequences caused by varying biophysical and biochemical properties of biomaterials is often not available with* in vitro* techniques. However, several 3D organ cultures have emerged, modeling the mechanical and biological microenvironment and interactions in prospective organs and implanted devices [64, 65].

Miniaturized fluid flow devices containing these 3D cultures, termed “organs-on-chips,” are the cutting edge alternative to animal models, allowing for high throughput examinations of a tissue or a tissue engineered construct. A microfluidic bone model was recently shown to monitor osteoblast behavior as well as formation of bone tissue and bacterial biofilm within an ink-jet printed poly(D,L-lactic-co-glycolic) acid construct containing biphasic calcium phosphate (BCP) and antibiotic nanoparticles [66]. While this proof of concept investigates just one prospective implant, several metrics were available to evaluate similar devices, such as calcium deposition, 3D tissue development, biofilm imaging, and osteoblast cell proliferation, migration, and development [66]. This class of technology has been useful in reproducing key physiological characteristics of an animal model, using* in vitro* cultures to more accurately predict* in vivo *consequences and success. In the design of novel biomimetic implantable materials, often laden with biologics, the ability to study tissue biology at high throughput affords the opportunity for accelerated progress in the field of bone tissue engineering and regeneration.

## 5. Conclusions

Briefly, we have covered recent advances in cell signaling biology, MSC isolation and purification, and biomaterials relevant to bone tissue engineering and regeneration. In the following special issue of BioMed Research International, we sought to incorporate these diverse areas of emphasis in order to reflect current trends in the field.
